# Body composition and testosterone in men: a Mendelian randomization study

**DOI:** 10.3389/fendo.2023.1277393

**Published:** 2023-11-27

**Authors:** Yoshihiro Ikehata, Tsuyoshi Hachiya, Takuro Kobayashi, Hisamitsu Ide, Shigeo Horie

**Affiliations:** ^1^ Department of Urology, Juntendo University, Graduate School of Medicine, Tokyo, Japan; ^2^ Department of Urology, Advanced informatics for genetic diseases, Juntendo University, Graduate School of Medicine, Tokyo, Japan

**Keywords:** BMI, fat mass, fat-free mass, body composition, exercise, testosterone levels, Mendelian randomization

## Abstract

**Background:**

Testosterone is an essential sex hormone that plays a vital role in the overall health and development of males. It is well known that obesity decreases testosterone levels, but it is difficult to determine the causal relationship between body composition and testosterone.

**Methods:**

To investigate potential causal associations between body composition and testosterone levels by a first time application of Mendelian randomization methods. Exposure variables in men included body composition (fat mass, fat-free mass, and body mass index). In addition to whole body fat and fat-free mass, we examined fat and fat-free mass for each body part (e.g., trunk, left arm, right arm, left leg and right leg) as exposures. Instrumental variables were defined using genome-wide association study data from the UK Biobank. Outcome variables in men included testosterone levels (total testosterone [TT], bioavailable testosterone [BT], and sex hormone-binding globulin [SHBG]). A one-sample Mendelian randomization analysis of inverse-variance weighted and weighted median was performed.

**Results:**

The number of genetic instruments for the 13 exposure traits related to body composition ranged from 156 to 540. Genetically predicted whole body fat mass was negatively associated with TT (β=-0.24, P=5.2×10-^33^), BT (β=-0.18, P=5.8×10-^20^) and SHBG (β=-0.06, P=8.0×10-^9^). Genetically predicted whole body fat-free mass was negatively associated with BT (β=-0.04, P=2.1×10-4), but not with TT and SHBG, after multiple testing corrections. When comparing the causal effect on testosterone levels, there was a consistent trend that the effect of fat mass was more potent than that of fat-free mass. There were no differences between body parts.

**Conclusion:**

These results show that reducing fat mass may increase testosterone levels.

## Introduction

Testosterone is an essential sex hormone that plays a vital role in males’ overall health and development. It is involved in maintaining the proper functioning of various systems and organs. Insufficient testosterone levels can lead to various signs and symptoms that may impact a males’ physical well-being and masculinity (1). Testosterone declines with aging, and a lower testosterone level is associated with muscle loss, fat gain, osteoporosis, cardiovascular problems, diabetes, hyperlipidemia, and cognitive decline in aging men (2). The syndrome, defined by low serum testosterone levels accompanied by characteristic symptoms, is known as late-onset hypogonadism (LOH) (2). Symptoms of LOH include general fatigue, diminished sexual desire, muscle weakness, erectile dysfunction (ED), poor concentration, insomnia, depression, headache, tinnitus, and diminished frequency of morning erections (3). In men aged 40-79, the incidence of symptomatic hypogonadism ranges from 2.1% to 5.7% (3, 4). Globally, the elderly population is increasing, particularly in developed countries, and as a result, the number of patients with LOH is expected to increase year-on-year. Therefore, the prevention of LOH, as well as the treatment of LOH symptoms, is becoming a more critical problem worldwide.

Testosterone levels are influenced by many factors. Higher testosterone levels are associated with daily physical activity (5), strength training, and aerobic exercise (6, 7). Conversely, obesity, diabetes, chronic obstructive pulmonary disease (COPD), nutritional deficiencies, such as zinc and vitamin D deficiency, as well as stress are associated with lower testosterone levels (8, 9). In particular, obesity in men is considered one of the most important factors responsible for low testosterone levels and is known as Male Obesity-associated Secondary Hypogonadism (MOSH) (10). The pathophysiological mechanisms of obesity-induced testosterone reduction are complex and multifactorial. Factors induced by obesity, such as the effects of systemic inflammation (11), increased aromatase activity (12), and leptin production (13), have all been suggested to interfere with testosterone production. In a study of 1,094 male patients with testosterone deficiency, the prevalences of metabolic syndrome was 69% (14). While little is known about the effects of muscle mass on testosterone, one study has found an association between muscle mass and testosterone levels, but this causal association is unclear due to the cross-sectional nature of the study (15). It is also known that accumulation of adipose tissue around the viscera, the internal organs of the body, is associated with the risk for development of cardiovascular and metabolic disease (16). Nevertheless, the association between fat or muscle distribution and testosterone levels is not yet known. In observational and interventional studies, it is difficult to assess the direct effect of body composition due to other confounding factors, so the causal relationship between body composition and testosterone levels remains to be elucidated (14).

To the best of the authors’ knowledge, this is the first time Mendelian randomization (MR) methods have been applied to investigate the potential causal associations between body composition and testosterone levels. MR can statistically elucidate the potential causal effects of an exposure variable on an outcome variable by using genetic variants as instrumental variables (IVs) (17). MR is like a natural randomized controlled trial (RCT) where genetic IVs, rather than doctors, randomly determine predispositions to certain traits. Just as RCTs, MR helps us infer causal relationships in observational data using genetic information. In this study, body composition (fat mass, fat-free mass, and body mass index [BMI]) in men was used as exposure variables, and IVs were defined using genome-wide association study (GWAS) data from the UK Biobank (18). In addition to whole body fat and fat-free mass, we examined fat and fat-free mass for each body part (e.g., trunk, left arm, right arm, left leg and right leg) as exposures. As outcome variables, testosterone levels (total testosterone [TT], bioavailable testosterone [BT], and sex hormone-binding globulin [SHBG]) in men were included in the analysis.

## Materials and methods

Methods of MR are based on association statistics rather than on individual-level data. There are able to estimate a causal effect of an exposure (*X*) on an outcome (*Y*), ${\hat{\beta }}_{XY}$ using multiple genetic variants $G_{i}\;\left(1\leq i\leq N\right)$ , where *N* denotes the number of genetic instruments and their effects on the exposure and outcome (denoted as $\beta_{GX}^{i}$ and $\beta_{GY}^{i}$ , respectively). When *N*=1, the causal effect of *X* on *Y* was estimated by dividing $\beta_{GY}$ by $\beta_{GX}$ (*i.e.*, ${\hat{\beta }}_{XY}=\beta_{GY}/\beta_{GX}$ ; referred to as ratio estimate or Wald estimate) under certain assumptions (19). The assumptions are: (i) the genetic variant is predictive of the exposure, (ii) the variant is independent of the outcome when conditioned on the exposure and any possible confounding factors, and (iii) the variant is independent of any confounding factors (20). When *N* ≥ 2, multiple Wald estimators were calculated from multiple genetic instruments ( ${\hat{\beta }}_{XY}^{i}=\beta_{GY}^{i}/\beta_{GX}^{i}$ ) and a meta-analysis was performed to obtain a final estimate of the causal effect. A well-used method for the meta-analysis of ${\hat{\beta }}_{XY}^{i}$ is the inverse-variance weighted (IVW) estimator (21), a weighted mean of individual Wald estimators. In IVW, the weight of the estimator ${\hat{\beta }}_{XY}^{i}$ is proportional to ${\left(\beta_{GX}^{i}\right)}^{2}/\sigma {\left(\beta_{GY}^{i}\right)}^{2}$ , and the weight of the estimator βXYi is proportional to $\left(\beta GXi\right)2\sigma \left(\beta_{GY}^{i}\right)$ 2 where σ(βGYi) is the standard error of the variance-outcome association estimate for the variant. If all genetic instruments satisfy the assumptions as mentioned earlier, there is no bias for the IVW estimate; however, bias is introduced if just one genetic variant is invalid (22). To relax these assumptions, several estimators, including weighted median (WM) (22) and MR-Egger (23), were developed. No bias is introduced for the WM if a large proportion of genetic variants are valid; therefore, this allows for invalid genetic instruments as long as they are not in the majority (22). The assumption of the MR-Egger is referred to as InSIDE (Instrument Strength Independent of Direct Effect), which states that the direct effect of the genetic instruments on the outcome, not through the exposure, is distributed independently of the strength of genetic effect on the exposure (*i.e.*, $\beta_{GX}$ ) (22, 23).

The inputs of the MR methods based on association statistics are variant-exposure and variant-outcome association effects (*i.e.*, $\beta_{GX}^{i}$ and $\beta_{GY}^{i}$ ), with standard errors on the same multiple genetic instruments. The variant-exposure and variant-outcome statistics can be retrieved from large-scale genome-wide association studies (GWAS) performed on various complex traits. When the variant-exposure and variant-outcome statistics are derived from separate studies (*i.e.*, populations from the two GWAS that do not overlap), a bias due to participant overlap can be avoided (24). A recent simulation study showed that the bias due to participant overlap was observed in the MR-Egger estimate but not in IVW and WM estimators if the single source of association statistics is based on a large number of participants (*n*>100,000), such as UK Biobank (25). Based upon this observation, the present study used the association statistics of exposures (13 traits related to body composition) and outcomes (three traits related to testosterone levels) derived from a single large-scale biobank study, the UK Biobank (18).

### Data source

**Figure 1:**
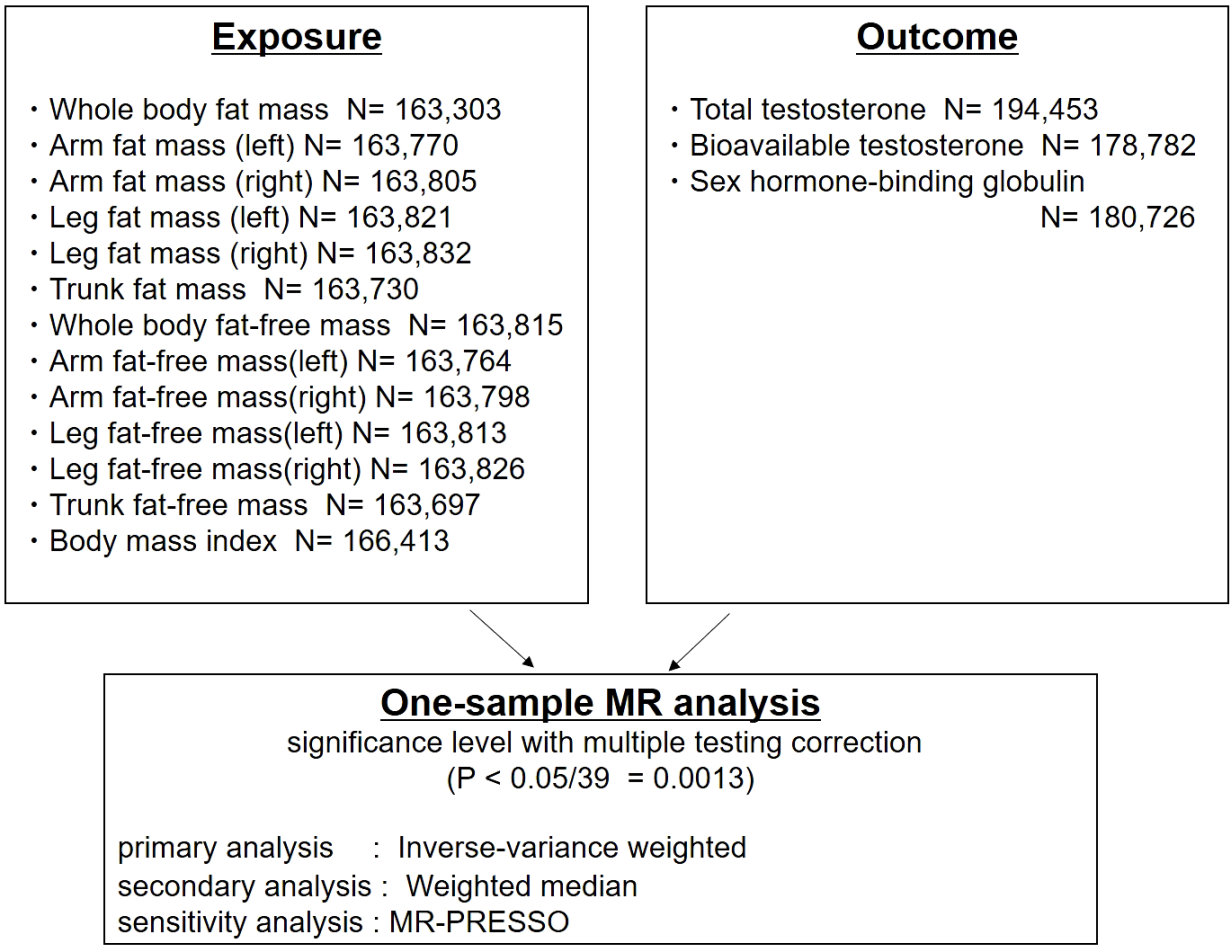
Study design: One-sample Mendelian randomization analysis of inverse-variance weighted and weighted median to evaluate the association between detailed body composition (body mass index [BMI], fat mass, fat-free mass), and testosterone levels (total testosterone, bioavailable testosterone, and sex hormone-binding globulin) in men. MR-PRESSO was used for the sensitivity analysis.

The study design is shown in **Figure 1**. To determine genetic instruments predictive of body composition, we utilized a large-scale GWAS of men of European ancestry from the UK Biobank (*n*=163,303 to 166,413). The UK Biobank is a large, population-based prospective cohort comprising linked health, hospital-record, and genetic data of individuals recruited across the UK (18). Body composition was measured by bioimpedance analysis (BIA) (Tanita BC418MA body composition analyser). BIA measures the electrical impedance of tissues in the body and estimates body composition based on this. Developments in BIA technology has now allowed for cost-efficient segmental body composition scans that estimate of the content of the trunk, arms and legs (26). Standing height was measured using a Seca 202 scale. Body mass index was calculated by dividing body weight and fat mass by height squared (kg/m^2^) (27).

Thirteen traits related to body composition were included (**Figure 1**). Publicly available genome-wide association statistics for the 13 traits were provided from Neale Lab, UK Biobank (imputed-v3, release 20180731). The genome-wide association statistics for the 13 traits included reference allele, alternate allele, β-coefficient, standard error of the β-coefficient, and *P*-values from up to 13,577,736 variants. We defined genetic instruments for the 13 traits based on genome-wide significant associations (*P*<5×10^−8^) after clumping for linkage disequilibrium at *R* ^2^<0.01 (based on the 1000 Genomes reference panel of European ancestry [*n*=503]) (28) using PLINK v1.90b6.8 (29).

A large-scale GWAS of testosterone levels (TT, BT, and SHBG) from men of European ancestry was conducted in a previous study based on UK Biobank data (*n*=178,782 to 194,453) (30). In this UK Biobank study, blood samples were collected at the initial visit. Total testosterone and SHBG-T (nmol/L) were measured by a one-step competitive analysis and a two-step sandwich immunoassay analysis (Beckman Coulter Unicel Dxl 800). Testosterone level bound to albumin (Alb-T; g/L) was measured by BCG analysis (Beckman Coulter AU5800). Bioavailable testosterone was calculated from TT, accounting for the concentration of SHBG-T and Alb-T using the Vermeulen equation (30). The genome-wide association statistics for TT, BT, and SHBG-T included 16,582,614 variants. We downloaded the genome-wide association statistics from the GWAS Catalog (TT, GCST90012113; BT, GCST90012103; and SHBG, GCST90012109). To perform the MR analyses, association statistics were extracted from the genetic instruments defined from the 13 body composition traits.

### Mendelian randomization analyses

Mendelian randomization analyses were performed to assess the effects of exposures (13 traits related to body composition) on outcomes (TT, BT, and SHBG-T). The causal association between exposure and outcome was estimated using IVW (21) and WM (22) estimators. The Steiger test was used to confirm the directionality of the effect on causality (31). We also used MR-PRESSO, MR-Egger, and Leave-one-out analysis for sensitivity analysis (32). All statistical tests were performed using the TwoSampleMR package version 0.5.6 and the R language version 4.2.1 (R Foundation for Statistical Computing, Vienna, Austria). The statistical testing was repeated 39 times (13 exposures × 3 outcomes), and therefore, two-sided *P* <0.0013 (=0.05/39) was considered statistically significant.

## Results

The number of genetic instruments used for the 13 exposure traits related to body composition ranged from 156 to 540 (**Table 1**; **Supplementary Tables S1–S13**). We calculated IVW and WM estimators to investigate potential causal relationships between exposures and outcomes (**Figure 1**). We also performed MR-PRESSO, MR-Egger, and Leave-one-out analysis as a sensitivity analysis. All results of the Steiger test were TRUE (*P*<0.001).

**Table 1:** Mendelian randomization analyses of body composition and testosterone levels: inverse-variance weighted.

Exposures		SNVs^a^	Total testosterone levels		Bioavailable testosterone levels		Sex hormone-binding globulin levels	
			IVW β	*P* ^b^	IVW β	*P* ^b^	IVW β	*P* ^b^
Whole body fat mass		170	-0.24	**5.17E-33**	-0.18	**5.78E-20**	-0.06	**8.02E-09**
			(-0.28 to -0.20)		(-0.22 to -0.14)		(-0.08 to -0.04)	
Arm fat mass	left	176	-0.23	**8.28E-21**	-0.17	**6.99E-22**	-0.06	**1.42E-05**
			(-0.27 to -0.18)		(-0.20 to -0.13)		(-0.08 to -0.03)	
	right	173	-0.23	**9.85E-21**	-0.16	**3.48E-19**	-0.06	**1.10E-05**
			(-0.28 to -0.18)		(-0.20 to -0.13)		(-0.08 to -0.03)	
Leg fat mass	left	163	-0.25	**4.94E-37**	-0.16	**1.73E-15**	-0.07	**3.36E-13**
			(-0.29 to -0.21)		(-0.20 to -0.12)		(-0.09 to -0.05)	
	right	156	-0.25	**1.97E-35**	-0.16	**1.43E-15**	-0.07	**1.44E-14**
			(-0.29 to -0.21)		(-0.20 to -0.12)		(-0.09 to -0.05)	
Trunk fat mass		162	-0.23	**1.51E-20**	-0.19	**3.35E-19**	-0.05	**1.19E-04**
			(-0.28 to 0.18)		(-0.23 to -0.15)		(-0.07 to -0.02)	
Whole body fat-free mass		458	-0.04	0.036	-0.04	**2.11E-04**	0	0.941
			(-0.07 to 0.00)		(-0.07 to -0.02)		(-0.02 to 0.02)	
Arm fat-free mass	left	389	-0.05	0.025	-0.04	0.004	-0.01	0.459
			(-0.09 to -0.01)		(-0.06 to -0.01)		(-0.03 to 0.01)	
	right	370	-0.05	0.024	-0.04	0.019	-0.01	0.369
			(-0.09 to -0.01)		(-0.06 to -0.01)		(-0.04 to 0.01)	
Leg fat-free mass	left	337	-0.08	**5.90E-04**	-0.08	**3.36E-08**	-0.01	0.403
			(-0.13 to -0.04)		(-0.11 to -0.05)		(-0.04 to 0.02)	
	right	339	-0.06	0.02	-0.07	**5.32E-06**	0	0.87
			(-0.11 to -0.01)		(-0.10 to -0.04)		(-0.03 to 0.02)	
Trunk fat-free mass		540	0	0.789	-0.02	0.068	0.01	0.485
			(-0.03 to 0.03)		(-0.04 to 0.00)		(-0.01 to 0.02)	
BMI		211	-0.24	**1.58E-30**	-0.13	**2.97E-17**	-0.08	**1.56E-11**
			(-0.28 to -0.20)		(-0.16 to -0.10)		(-0.10 to -0.05)	

BMI, body mass index; IVW, inverse-variance weighted; SNVs, single-nucleotide variations.

a Number of SNVs retained for this analysis.

b P less than 0.001 appears in bold.

### Association of genetically predicted fat mass and testosterone

**Figure 2:**
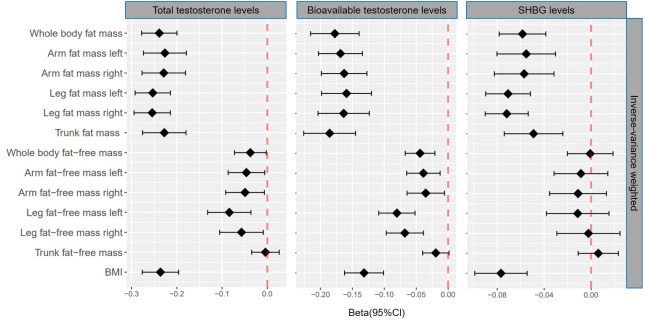
βcoefficients with 95% CIs of inverse-variance weighted for the effect of one unit increase in fat mass, fat-free mass, and body mass index (BMI) on testosterone levels (total testosterone, bioavailable testosterone, and sex hormone-binding globulin) in men. SHBG, sex hormone-binding globulin.

**Figure 3:**
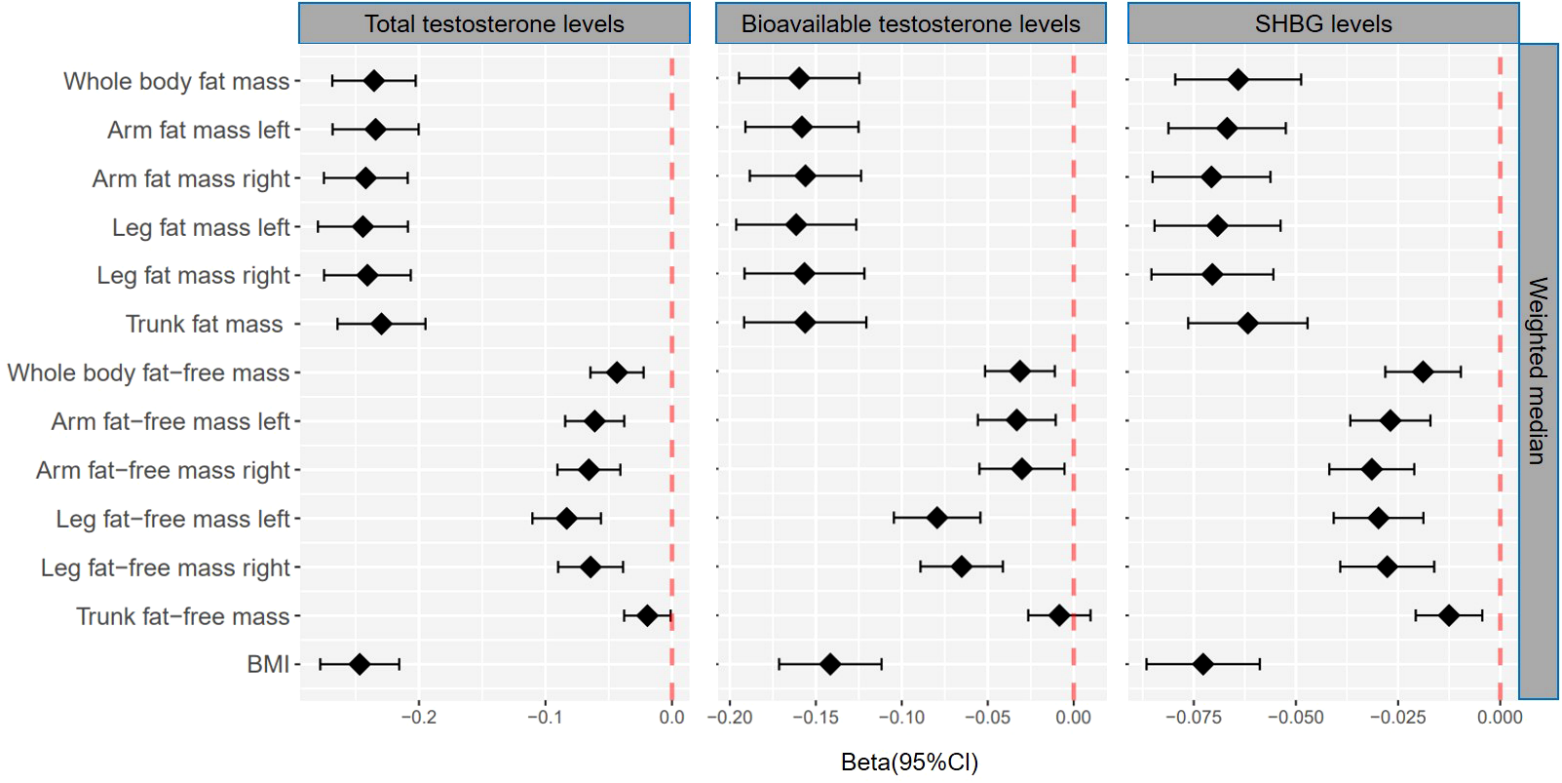
βcoefficients with 95% CIs of weighted median for the effect of one unit increase in fat mass, fat-free mass, and body mass index (BMI) on testosterone levels (total testosterone, bioavailable testosterone, and sex hormone-binding globulin) in men. SHBG, sex hormone-binding globulin.

Genetically predicted whole body fat mass was negatively associated with TT (IVW: β=-0.24 [95% CI: -0.28 to -0.20], *P*=5.2×10^-33^; WM: β=-0.24 [95% CI: -0.27 to -0.20], *P*=1.7×10^-44^), BT (IVW: β=-0.18 [95% CI: -0.22 to -0.14], *P*=5.8×10^-20^; WM: β=-0.16 [95% CI: -0.19 to -0.12], *P*=3.7×10^-19^) and SHBG (IVW: β=-0.06 [95% CI: -0.08 to -0.04], *P*=8.0×10^-9^; WM: β=-0.06 [95% CI: -0.08 to -0.05], *P*=3.3×10^-16^). Similar associations were observed when fat mass for individual body parts was used (*i.e.*, arm fat mass [left], arm fat mass [right], leg fat mass [left], leg fat mass [right], and trunk fat mass) as exposure and there were no differences between body parts (**Figures 2**, **3**, **Tables 1**, **2**).

**Table 2:** Mendelian randomization analyses of body composition and testosterone levels: weighted median.

Exposures		SNVs^a^	Total testosterone levels		Bioavailable testosterone levels		Sex hormone-binding globulin levels	
			WM β	*P* ^b^	WM β	*P* ^b^	WM β	*P* ^b^
Whole body fat mass		170	-0.24	**1.66E-44**	-0.16	**3.70E-19**	-0.06	**3.32E-16**
			(-0.27 to -0.20)		(-0.19 to -0.12)		(-0.08 to -0.05)	
Arm fat mass	left	176	-0.23	**1.51E-41**	-0.16	**4.68E-21**	-0.07	**7.52E-20**
			(-0.27 to -0.20)		(-0.19 to -0.13)		(-0.08 to -0.05)	
	right	173	-0.24	**1.57E-46**	-0.16	**3.57E-21**	-0.07	**7.96E-22**
			(-0.28 to -0.21)		(-0.19 to -0.12)		(-0.09 to -0.06)	
Leg fat mass	left	163	-0.24	**2.22E-41**	-0.16	**1.30E-19**	-0.07	**1.50E-18**
			(-0.28 to -0.21)		(-0.20 to -0.13)		(-0.08 to -0.05)	
	right	156	-0.24	**3.83E-43**	-0.16	**1.32E-18**	-0.07	**2.16E-20**
			(-0.28 to -0.21)		(-0.19 to -0.12)		(-0.09 to -0.06)	
Trunk fat mass		162	-0.23	**2.16E-38**	-0.16	**7.40E-18**	-0.06	**1.13E-16**
			(-0.26 to 0.19)		(-0.19 to -0.12)		(-0.08 to -0.05)	
Whole body fat-free mass		458	-0.04	5.00E-05	-0.03	0.003	0.02	**6.21E-05**
			(-0.06 to -0.02)		(-0.05 to -0.01)		(-0.03 to -0.01)	
Arm fat-free mass	left	389	-0.06	**2.90E-07**	-0.03	0.004	-0.03	**7.82E-08**
			(-0.08 to -0.04)		(-0.06 to -0.01)		(-0.04 to -0.02)	
	right	370	-0.07	**2.20E-07**	-0.03	0.017	-0.03	**3.05E-09**
			(-0.09 to -0.04)		(-0.05 to -0.01)		(-0.04 to -0.02)	
Leg fat-free mass	left	337	-0.08	**1.67E-09**	-0.08	**6.04E-10**	-0.03	**1.03E-07**
			(-0.11 to -0.06)		(-0.10 to -0.05)		(-0.04 to -0.02)	
	right	339	-0.06	**8.83E-07**	-0.07	**9.72E-08**	-0.03	**2.44E-06**
			(-0.09 to -0.04)		(-0.09 to -0.04)		(-0.04 to -0.02)	
Trunk fat-free mass		540	-0.02	0.038	-0.01	0.367	-0.01	0.003
			(-0.04 to 0.00)		(-0.03 to 0.01)		(-0.02 to 0.00)	
BMI		211	-0.25	**2.99E-54**	-0.14	**1.17E-20**	-0.07	**8.71E-25**
			(-0.28 to -0.22)		(-0.17 to -0.11)		(-0.09 to -0.06)	

BMI, body mass index; WM, weighted median; SNVs, single-nucleotide variations.

a Number of SNVs retained for this analysis.

b P less than 0.001 appears in bold.

### Association of genetically predicted fat-free mass and testosterone

According to IVW, genetically predicted whole body fat-free mass was negatively associated with BT (β=-0.04 [95% CI: -0.07 to -0.02], *P*=2.1×10^-4^) but not with TT and SHBG after multiple testing corrections (**Table 1**). In contrast, WM suggested that genetically predicted whole body fat-free mass was negatively associated with TT (β=-0.04 [95% CI: -0.06 to -0.02], *P*=5.0×10^-5^) and SHBG (β=-0.02 [95% CI: -0.03 to -0.01], *P*=6.2×10^-5^), but not with BT after multiple testing corrections (**Table 2**). When fat-free mass for individual body parts was used as exposure, three (IVW) and 10 (WM) significant exposure-outcome pairs were suggested. There were no differences between body parts (**Tables 1**, **2**).

When comparing the causal effect on testosterone levels, a consistent trend was found – the effect of fat mass was stronger than that of fat-free mass (**Figures 2**, **3**). For example, the absolute value of β- coefficients of whole body fat mass were consistently more significant than that of whole body fat-free mass (IVW: β_TT_, -0.24 *vs*. -0.04; β_BT_, -0.18 *vs*. -0.04; and β_SHBG_, -0.06 *vs*. 0.00, WM: β_TT_, -0.24 *vs*. -0.04; β_BT_, -0.16 *vs*. -0.03; and β_SHBG_, -0.06 *vs*. -0.02). Similar trends were observed when fat mass and fat-free mass were compared for individual body parts.

### Association of genetically predicted BMI and testosterone

Genetically predicted BMI was negatively associated with TT (IVW: β=-0.24 [95% CI: -0.28 to -0.20], *P*=1.6×10^-30^; WM: β=-0.25 [95% CI: -0.28 to -0.22], *P*=3.0×10^-54^), BT (IVW: β=-0.13 [95% CI: -0.16 to -0.10], *P*= 3.0×10^-17^; WM: β=-0.14 [95% CI: -0.17 to -0.11], *P*=1.2×10^-20^) and SHBG (IVW: β=-0.08 [95% CI: -0.10 to -0.05], *P*=1.6×10^-11^; WM: β=-0.07 [95% CI: -0.09 to -0.06], *P*=8.7×10^-25^) (**Tables 1**, **2**). The comparison of a causal effect on testosterone levels revealed that the absolute value of β-coefficients for BMI was equivalent to a fat mass of the whole body or each body part. It was more significant than fat-free mass of the whole body or each body part (**Figures 2**, **3**).

### Sensitivity analysis

Similar results were observed using MR-PRESSO, while MR-Egger showed an attenuated causal relationship between each exposure and testosterone levels (**Supplementary Figures S1, S2**, **Supplementary Tables S14, S15**). Leave-one-out analysis also showed that individual SNPs did not affect the overall estimates (**Supplementary Figures S3–5**).

## Discussion

It is well known that obesity decreases testosterone levels, but it is difficult to determine the causal relationship between body composition and testosterone. This is the first study to investigate potential causal associations of detailed body composition parameters with testosterone levels based on large-scale GWAS data and MR analyses. These results showed that genetically higher fat mass was associated with lower testosterone levels in men of European ancestry but that genetically predicted fat-free mass was either only weakly associated or not statistically associated with testosterone levels. There were no differences between body parts. With the exception of MR-Egger, which may give a biased estimate in a single-sample MR analysis (25), different MR methods consistently supported our findings. Therefore, this MR study suggests that fat mass lowers testosterone levels, while muscle mass may not be related to testosterone levels.

Various observational studies have reported an association between obesity and low testosterone, but the mechanism is multifactorial and possibly bidirectional, and the exact causal relationship is largely unknown (33, 34). Our findings that fat mass lowers testosterone support the direction that fat lowers testosterone. Indeed, there have been reports of post-operative increases in testosterone in patients undergoing bariatric surgery (35, 36). Grossmann M et al. said that weight loss, whether dietary or surgical, also leads to significant increases in total testosterone in proportion to the amount of weight loss, particularly in morbidly obese men (37). These reports are consistent with the results of the present study, which suggest that fat itself may affect low testosterone. There is a negative correlation between BMI and SHBG, and the main cause of lower TT in obesity is lower SHBG. In people with severe obesity, TT is not only decreased but BT also. Decreased BT is not, however, associated with increased luteinizing hormone (LH), which may be due to decreased gonadotropin-releasing hormone (GnRH)/LH secretion (38). Obesity in men can lead to reduced testosterone levels due to several mechanisms. Increased aromatase activity in fat cells converts testosterone to estradiol, suppressing the hypothalamic–pituitary–thyroid (HPT) axis (39). Elevated leptin, a hormone from fat cells, also impacts this axis, decreasing testosterone (40, 41). Additionally, obesity can cause leptin resistance and impact Leydig cell responsiveness (40). Obesity-related inflammation might further suppress testosterone production, and complications like obstructive sleep apnea, which reduces REM sleep and induces nocturnal hypoxia, further contribute to this reduction (12, 42).

Fat-free mass includes mainly muscle, bone, and water but is generally related to muscle mass. The effect of muscle mass on testosterone is not well understood. Strength training, such as deadlifts and squats, which use larger muscles, has been associated with significant increases in testosterone levels (43). While it has been reported that muscle mass does not affect the degree of testosterone elevation immediately after training (44), elevated testosterone levels immediately after muscle training and aerobic exercise have been reported (43), and attributed to, reduced plasma volume, adrenergic stimulation (45), lactate-stimulated secretion (46), potential effects on testosterone synthesis, and secretory capacity of Leydig cells in the testis (47). On the other hand, the long-term effects of muscle training and aerobic exercise on testosterone are controversial (43). While some studies report long-term testosterone elevation (45, 46), others report no changes (48). Effects may vary according to exercise intensity or age. The conventional phenomenon that muscle training and aerobic exercise raise testosterone levels may be due to a biochemical effect of the activities themselves, and increasing muscle mass itself may have no direct effect on testosterone levels.

This study has several limitations. Firstly, this was not an interventional study. Secondly, body composition in the GWAS used in this study was measured by BIA, not Dual-energy X-ray Absorptiometry (DXA). DXA is more accurate for the assessment of body composition. In addition, this MR analysis was performed in a European population, and it is unknown whether similar findings would be present in other racial groups. There is a need for large-scale GWAS in Asian and African populations.

In conclusion, we found a negative association between genetically predicted fat mass and testosterone levels and a weak or no association between fat-free mass and testosterone levels in men of European ancestry. This MR study suggests that muscle mass itself may not be related to testosterone; however, the effect of fat mass can lower testosterone levels. Therefore, reducing fat mass may be necessary to maintain or elevate testosterone levels. Further interventional and MR studies are required, including GWAS in different racial populations.

## Data availability statement

The original contributions presented in the study are included in the article/**Supplementary Material**. Further inquiries can be directed to the corresponding author.

## Ethics statement

Ethical approval was not required for the studies involving humans because The studies involving human participants were reviewed and approved by the each participating study from its institutional review board. The studies were conducted in accordance with the local legislation and institutional requirements. The human samples used in this study were acquired from Written informed consent was not provided because the original data was not collected for this manuscript. Written informed consent to participate in this study was not required from the participants or the participants’ legal guardians/next of kin in accordance with the national legislation and the institutional requirements.

## Author contributions

YI: Conceptualization, Data curation, Formal analysis, Investigation, Resources, Validation, Writing – original draft, Writing – review & editing. TH: Data curation, Formal analysis, Investigation, Resources, Validation, Visualization, Writing – review & editing. TK: Investigation, Validation, Writing – review & editing. HI: Project administration, Supervision, Writing – review & editing. SH: Conceptualization, Project administration, Supervision, Writing – review & editing.
