# Supplementary material for: Body composition and testosterone in men: a Mendelian randomization study
Source: Front Endocrinol (Lausanne). 2023 Nov 27;14:1277393. doi: 10.3389/fendo.2023.1277393 (PMC10711270; doi:10.3389/fendo.2023.1277393)
Supplement: Supplementary file 2 [file DataSheet_1.docx]

Supplementary Material

Body composition and testosterone in men: A Mendelian randomization study

Yoshihiro Ikehata, Tsuyoshi Hachiya, Takuro Kobayashi, Hisamitsu Ide, Shigeo Horie*

*Correspondence: [shorie@juntendo.ac.jp](mailto:shorie@juntendo.ac.jp)

**1 Supplementary Figure and Tables**

- 1. **Supplementary Figure**


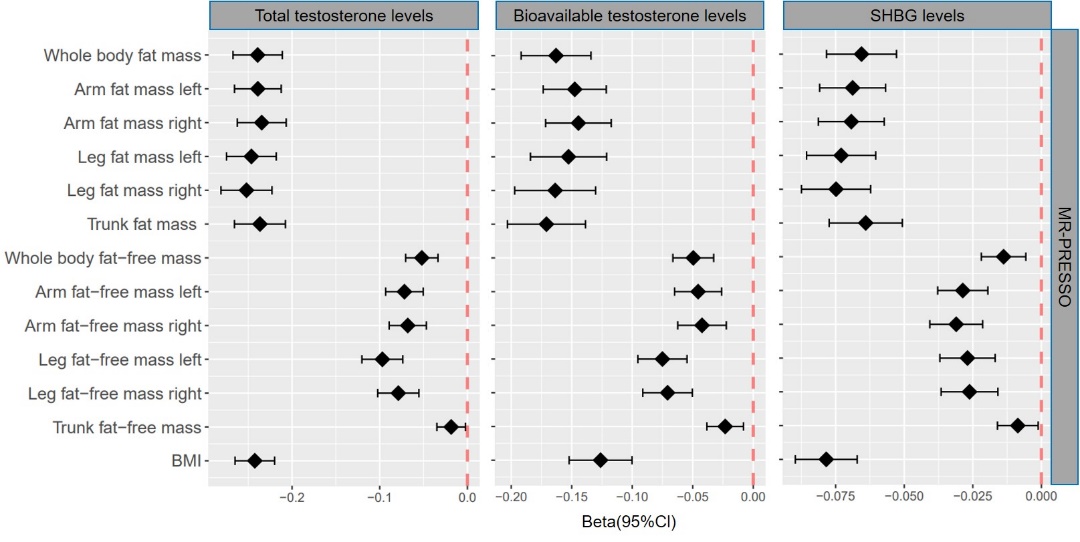


**Supplementary Figure 1.**

β coefficients with 95% CIs of MR-PRESSO for the effect of one unit increase in fat mass, fat-free mass, and body mass index (BMI) on testosterone levels (total testosterone, bioavailable testosterone, and sex hormone-binding globulin) in men. Abbreviation: SHBG, sex hormone-binding globulin.


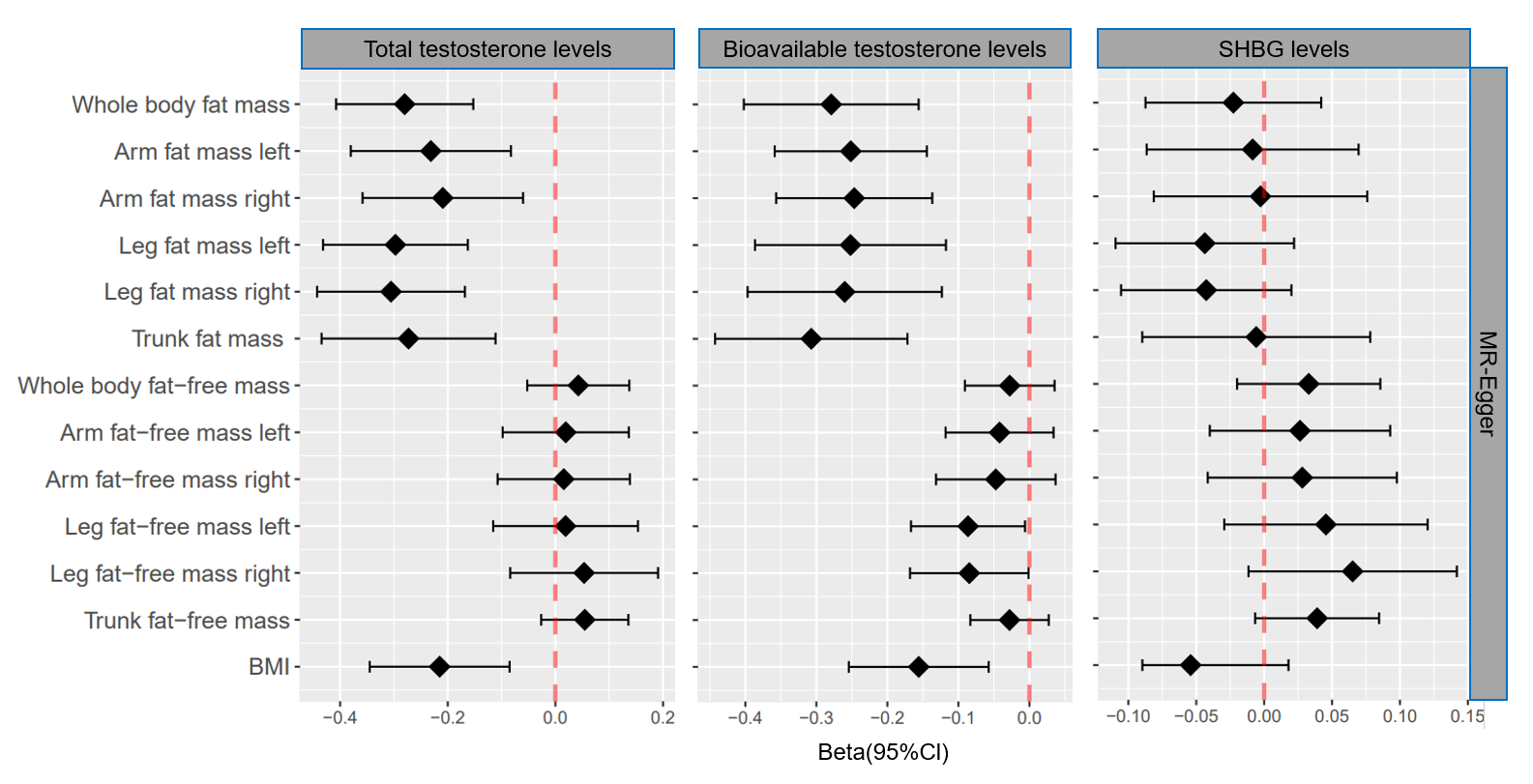


**Supplementary Figure 2.**

β coefficients with 95% CIs of MR-Egger for the effect of one unit increase in fat mass, fat-free mass, and body mass index (BMI) on testosterone levels (total testosterone, bioavailable testosterone, and sex hormone-binding globulin) in men. Abbreviation: SHBG, sex hormone-binding globulin.


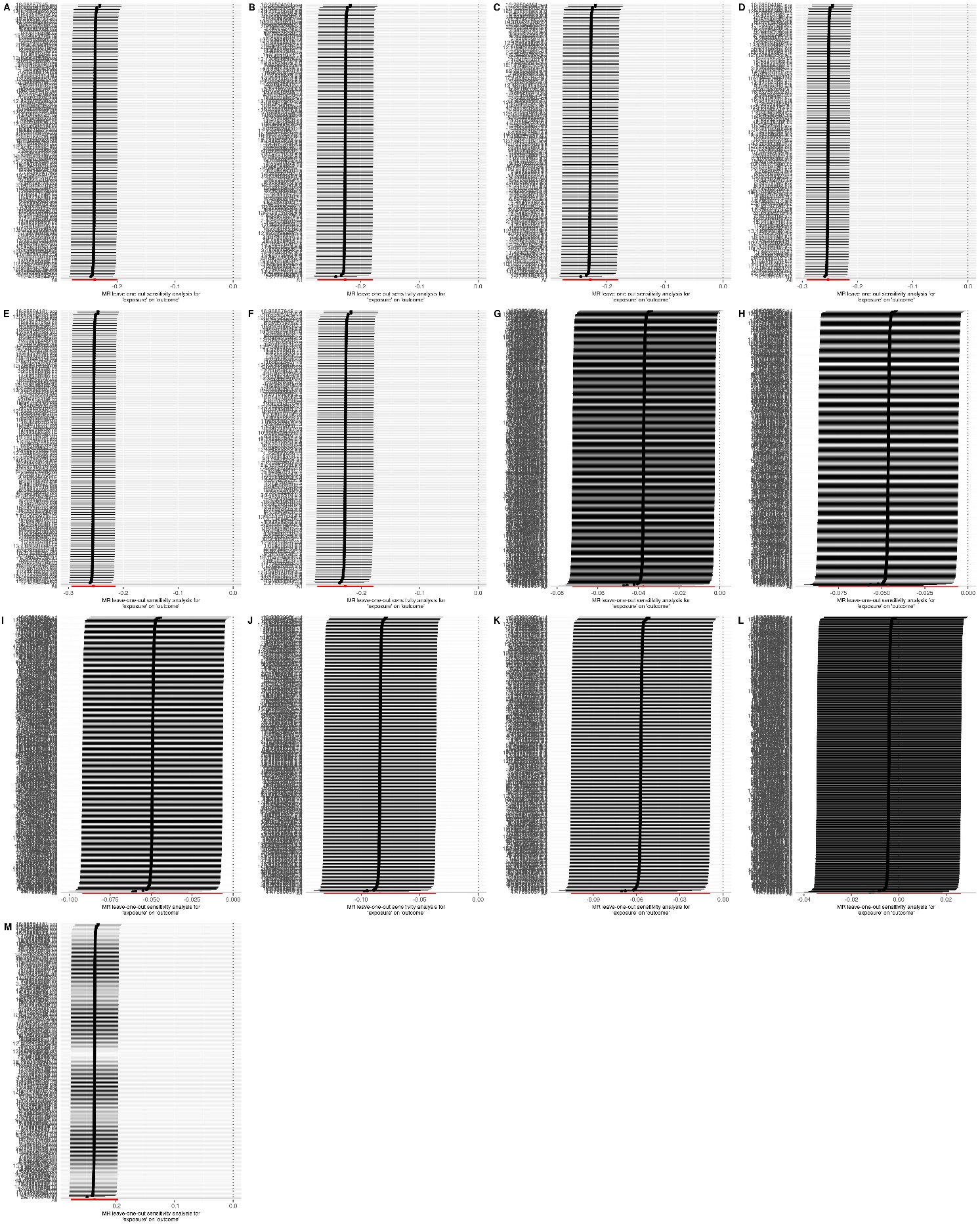


**Supplementary Figure 3.**

Leave-one-out plots for the causal relationship between body composition and total testosterone (TT). (A) MR leave-one-out sensitivity analysis for whole body fat mass on TT. (B) MR leave-one-out sensitivity analysis for arm fat mass (left) on TT. (C) MR leave-one-out sensitivity analysis for arm fat mass (right) on TT. (D) MR leave-one-out sensitivity analysis for leg fat mass (left) on TT. (E) MR leave-one-out sensitivity analysis for leg fat mass (right) on TT. (F) MR leave-one-out sensitivity analysis for trunk fat mass on TT. (G) MR leave-one-out sensitivity analysis for whole body fat-free mass on TT. (H) MR leave-one-out sensitivity analysis for arm fat-free mass (left) on TT. (I) MR leave-one-out sensitivity analysis for arm fat-free mass (right) on TT. (J) MR leave-one-out sensitivity analysis for leg fat-free mass (left) on TT. (K) MR leave-one-out sensitivity analysis for leg fat-free mass (right) on TT. (L) MR leave-one-out sensitivity analysis for trunk fat-free mass on TT. (M) MR leave-one-out sensitivity analysis for body mass index on TT. Abbreviations: MR, Mendelian randomization


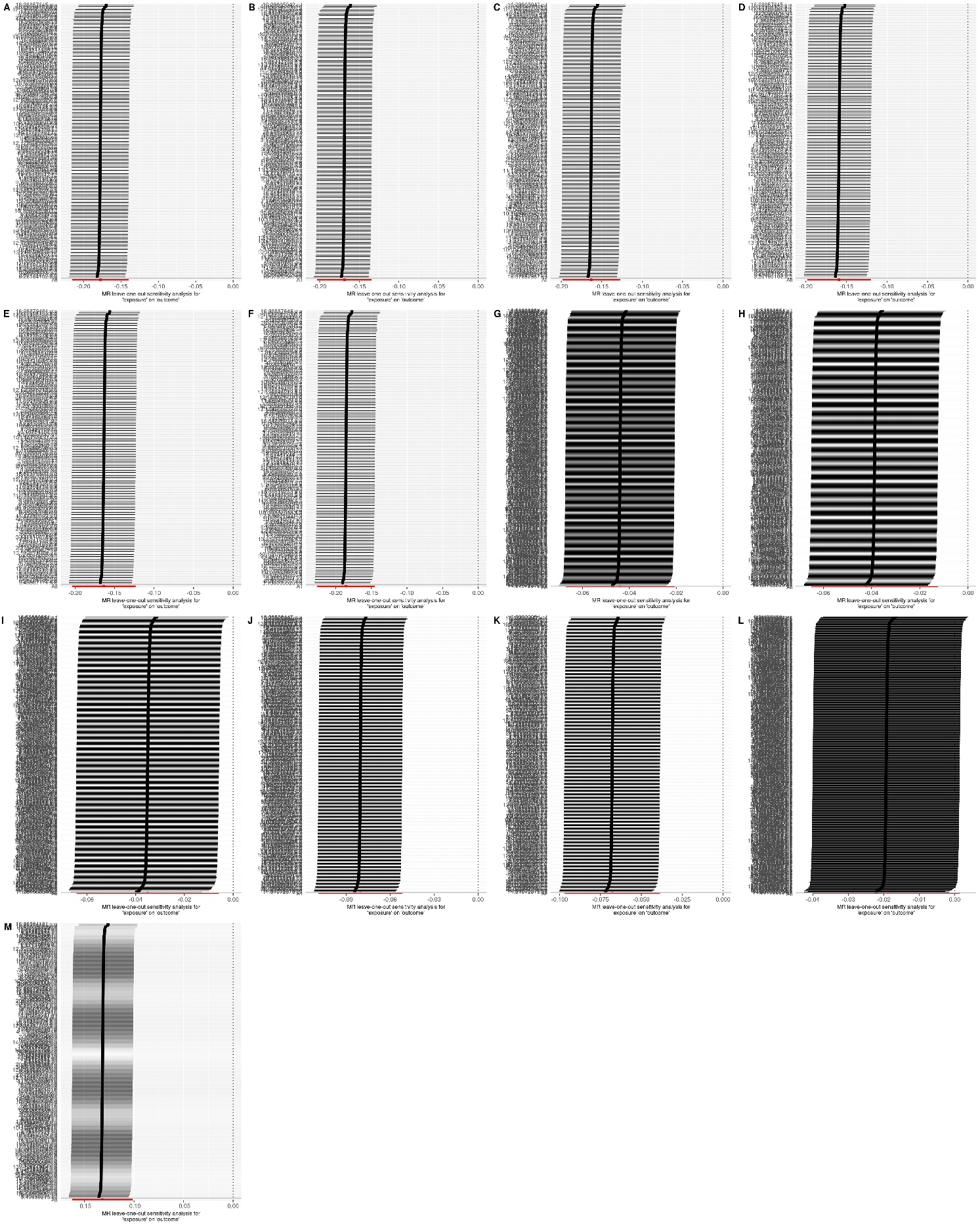


**Supplementary Figure 4.**

Leave-one-out plots for the causal relationship between body composition and bioavailable testosterone (BT). (A) MR leave-one-out sensitivity analysis for whole body fat mass on BT. (B) MR leave-one-out sensitivity analysis for arm fat mass (left) on BT. (C) MR leave-one-out sensitivity analysis for arm fat mass (right) on BT. (D) MR leave-one-out sensitivity analysis for leg fat mass (left) on BT. (E) MR leave-one-out sensitivity analysis for leg fat mass (right) on BT. (F) MR leave-one-out sensitivity analysis for trunk fat mass on BT. (G) MR leave-one-out sensitivity analysis for whole body fat-free mass on BT. (H) MR leave-one-out sensitivity analysis for arm fat-free mass (left) on BT. (I) MR leave-one-out sensitivity analysis for arm fat-free mass (right) on BT. (J) MR leave-one-out sensitivity analysis for leg fat-free mass (left) on BT. (K) MR leave-one-out sensitivity analysis for leg fat-free mass (right) on BT. (L) MR leave-one-out sensitivity analysis for trunk fat-free mass on BT. (M) MR leave-one-out sensitivity analysis for body mass index on BT. Abbreviations: MR, Mendelian randomization


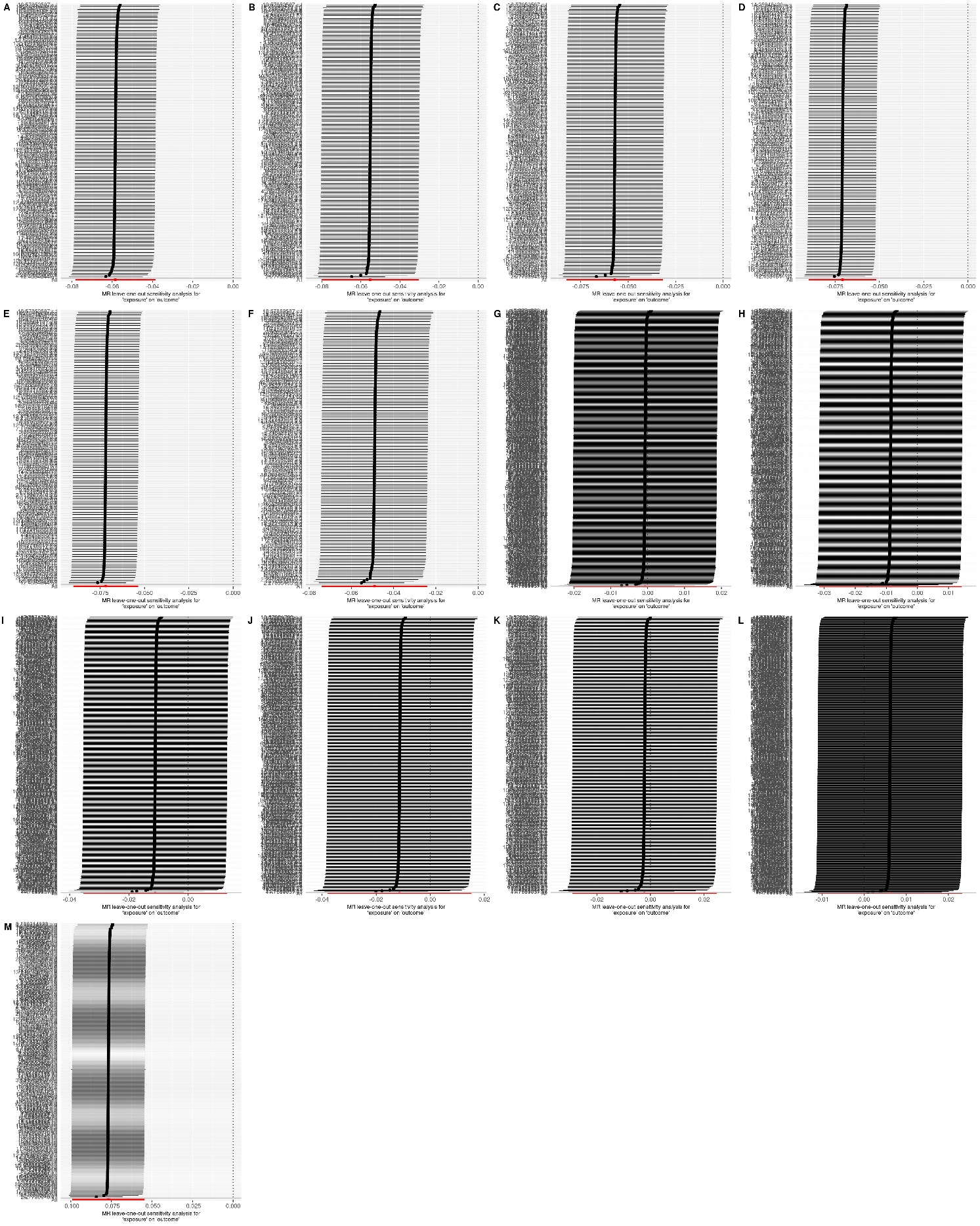


**Supplementary Figure 5.**

Leave-one-out plots for the causal relationship between body composition and sex hormone-binding globulin (SHBG). (A) MR leave-one-out sensitivity analysis for whole body fat mass on SHBG. (B) MR leave-one-out sensitivity analysis for arm fat mass (left) on SHBG. (C) MR leave-one-out sensitivity analysis for arm fat mass (right) on SHBG. (D) MR leave-one-out sensitivity analysis for leg fat mass (left) on SHBG. (E) MR leave-one-out sensitivity analysis for leg fat mass (right) on SHBG. (F) MR leave-one-out sensitivity analysis for trunk fat mass on SHBG. (G) MR leave-one-out sensitivity analysis for whole body fat-free mass on SHBG. (H) MR leave-one-out sensitivity analysis for arm fat-free mass (left) on SHBG. (I) MR leave-one-out sensitivity analysis for arm fat-free mass (right) on SHBG. (J) MR leave-one-out sensitivity analysis for leg fat-free mass (left) on SHBG. (K) MR leave-one-out sensitivity analysis for leg fat-free mass (right) on SHBG. (L) MR leave-one-out sensitivity analysis for trunk fat-free mass on SHBG. (M) MR leave-one-out sensitivity analysis for body mass index on SHBG. Abbreviations: MR, Mendelian randomization

- 1. **Supplementary Tables**

**Supplementary Table 1.** 170 Whole body fat mass-related variants defined using genome-wide association study data from the UK Biobank.

**Supplementary Table 2.** 176 Arm fat mass (left)-related variants defined using genome-wide association study data from the UK Biobank.

**Supplementary Table 3.** 173 Arm fat mass (right)-related variants defined using genome-wide association study data from the UK Biobank.

**Supplementary Table 4.** 163 Leg fat mass (left)-related variants defined using genome-wide association study data from the UK Biobank.

**Supplementary Table 5.** 156 Leg fat mass (right)-related variants defined using genome-wide association study data from the UK Biobank.

**Supplementary Table 6.** 162 Trunk fat mass-related variants defined using genome-wide association study data from the UK Biobank.

**Supplementary Table 7.** 458 Whole body fat-free mass-related variants defined using genome-wide association study data from the UK Biobank.

**Supplementary Table 8.** 389 Arm fat-free mass (left)-related variants defined using genome-wide association study data from the UK Biobank.

**Supplementary Table 9.** 370 Arm fat-free mass (right)-related variants defined using genome-wide association study data from the UK Biobank.

**Supplementary Table 10.** 337 Leg fat-free mass (left)-related variants defined using genome-wide association study data from the UK Biobank.

**Supplementary Table 11.** 339 Leg fat-free mass (right)-related variants defined using genome-wide association study data from the UK Biobank.

**Supplementary Table 12.** 540 Trunk fat-free mass-related variants defined using genome-wide association study data from the UK Biobank.

**Supplementary Table 13.** 211 body mass index-related variants defined using genome-wide association study data from the UK Biobank.

**Supplementary Table 14.** Mendelian randomization results of MR-PRESSO.

Abbreviations: BMI, body mass index; SNVs, single-nucleotide variations.

^a^Number of SNVs retained for this analysis.

^b^*P* less than 0.001 appears in bold.

**Supplementary Table 15.** Mendelian randomization results of MR-Egger.

Abbreviations: BMI, body mass index; SNVs, single-nucleotide variations.

^a^Number of SNVs retained for this analysis.

^b^*P* less than 0.001 appears in bold.
